# 

**Published:** 1882-10

**Authors:** 


### CONTENTS:

PAGE.

ORIGINAL COMMUNICATIONS:

I. The Present Status of Antiseptic Surgery. By Roswell Park, M.D. ........ 338
II. The Yellow Oxide of Mercury. By W. W. Seely, M.D. ........ 351
III. Summary of Monthly Reports of Medical Department of Southern Illinois Penitentiary. By H. Z. Gill, M.D. ........ 356
IV. Rupture of Left Kidney. By R. N. Isham, M.D. ........ 364
V. Report of Two Cases of Traumatic Tetanus, with Recovery. By F. W. Stebalts, M.D. ........ 367

HOSPITAL REPORTS:

VI. Cook County Hospital Case of Tetanus Treated by Nerve Stretching; Recovery ........ 369

SOCIETY REPORTS:

VII. Chicago Medical Society ........ 373

REVIEWS AND BOOK NOTICES ........ 376

EDITORIAL:

A "Rod" in Pickle for the Bacillus Tuberculosis ........ 408

ORIGINAL TRANSLATIONS:

A Case of Myelitis, consequent upon Meningitis Cerebro-Spinalis, with Double Sensation, and its Graphic Representation ........ 412

PAGE

On Resection of the Stomach ........ 418
Amaurosis Chinica ........ 420
Pyometra caused by an Adenoma occluding the Cervix Uteri ........ 421
Double Peripleuritis with Suppurative Mediastinitis ........ 422
Syphilis of the Brain ........ 423
A certain form of Muscular Atrophy Peculiar to the Lower Extremities ........ 424
Rupture of an Umbilical Hernia, with prolapsus of Intestines ........ 425
Vaccination in Germany ........ 426
Anæsthesia of Plants ........ 427
Interstitial Chronic Hepatitis ........ 428
Contraction of the Orifice of the Pulmonary Artery without Tuberculosis ........ 428
The Bacilli of Tubercle ........ 429
Nitrous Oxide ........ 429

SELECTIONS:

Some Fallacies of the Bioplasm Theory. A Reply to Dr. Heitzmann by Lester Curtis, M.D. ........ 431
On the Treatment of Dorso-Lumbar Abscess by Means of Free Incisions and Subsequent Carbolized Injections. By Norman H. Chapman, M.D. ........ 438

ITEMS ........ 368, 372, 375, 407, 411, 443, 444

ANNOUNCEMENTS FOR THE MONTH ........ 448

NOTICE TO CORRESPONDENTS ........ advts

